# Reviewers BJCVS 31.6

**Published:** 2016

The Brazilian Journal of Cardiovascular Surgery (BJCVS) is grateful for the reviewers, listed below, which collaborated in this edition. Without their work would be impossible to keep the high scientific standard of our journal.

Carlos Piccinato

Eduardo Augusto Victor Rocha

Eduardo Keller Saadi

Francisco Costa

Gibran Roder Feguri

Giovani Jose Dal Poggetto Molinari

Guilherme Agreli

Hélcio Giffhorn

João Carlos Ferreira Leal

José Glauco Lobo Filho

Nelson Hossne

Otoni Gomes

Pablo Maria Alberto Pomerantzeff

Paulo Brofman

Ricardo Ribeiro Dias

Stevan Krieger Martins

